# Using eye-tracking technology in Neuromarketing

**DOI:** 10.22336/rjo.2023.2

**Published:** 2023

**Authors:** Consuela-Mădălina Gheorghe, Victor Lorin Purcărea, Iuliana-Raluca Gheorghe

**Affiliations:** * Department of Marketing and Medical Technology, “Carol Davila” University of Medicine and Pharmacy, Bucharest, Romania

**Keywords:** eye tracking, Neuromarketing, gaze movements, visual elements

## Abstract

The aim of this review is to highlight the importance and benefits of eye tracking in Neuromarketing, by analyzing research papers and articles from international databases such as Scopus, PubMed, Elsevier, Springer and Science Direct. After analyzing some of the most representative publications related to eye tracking in Neuromarketing, we have concluded that it can be considered one of the most important tools in determining the consumers’ intent to buy medical products and services.

In addition, with a mixture between standard Marketing Research methods and the eye-tracking technology, the researchers can get insight into unconscious factors that influence the consumers’ choices and preferences.

In conclusion, neuromarketing can prevent the waste of money spent on ineffective marketing campaigns.

## Introduction

For many decades, eye tracking has been used to investigate gaze behavior in the population, by showing the way different stimuli are processed. The processed stimuli may be images, colors, and even persons. Such a system is also used in Neuromarketing, which is an emergent field that combines Behavioral Psychology, Economics and Consumer Neuroscience. Thus, with a mixture between standard Marketing Research methods and the eye-tracking technology, the researchers would be able to get insight into unconscious factors that influence the consumers’ choices and preferences. Also, it should be considered that, from an ethical standpoint, Neuromarketing is a controversial field and an explicit informed consent should be provided. However, by using eye tracking, marketing experts can assess the unconscious preferences of prospective consumers. 

## Defining Neuromarketing

Neuromarketing was first used in 2002 by Ale Smidts, who defined it as “the study of the cerebral mechanism to understand the consumer’s behavior in order to improve the marketing strategies” [**1**]. Nowadays, the field is considered a combination between Behavioral Psychology, Economics and Consumer Neuroscience [**2**]. 

To understand the choices and triggers or preferences of consumers, Neuromarketing uses many techniques, among which, the most important and least intrusive (from an ethical viewpoint) is eye tracking [**2**]. 

Among the advantages of neuromarketing are the tools that are used to spot stimuli and indications, which disclose consumer behavior that cannot be observed with good visual inspection. 

Neuromarketing is the field that can measure attention, emotions, motivation, senses and memory of the consumers, among which, emotions are the only ones that can appear without awareness. Motivation seems to be what shapes the consumer’s wants and likes. Attention is also regarded as an important process, and is of two types: bottom up (accidentally looking for something) and top-down (looking for something particular). The bottom-up attention deals with things that automatically draw the consumers’ attention, such as movement, brightness, etc. [**3**]. It has been proved that by modifying the visual appearance of a medical product or the advertisement for a service, consumers are more likely to look at it and engage in buying [**4**]. 

Moreover, since 95% of the decision-making process occurs at an unconscious level, most people are unable to explain their purchase decisions, but, to overcome this limitation, specialists began to understand these processes that happen in the minds of the consumers, by using neuroscientific techniques [**5**]. One of the main techniques is eye tracking, which is based on the visual behavior (e.g., fixation point, gaze and pupil dilation); visual attention mechanisms; and consumer engagement by showing interest at a visual level [**6**]. Thus, eye tracking is considered a very important part of Neuromarketing and experimental economics research [**7**]. 

## Defining the eye-tracking technique

The first to mention eye tracking was Louis Emile Javal, who observed, in 1879, that reading does not imply a smooth sweeping of the eyes along the text, as it was assumed, but a series of short stops and saccades. 

At the same time, Edmund Huey built the first eye tracker that used a sort of contact lens with a hole for the pupil. The lens was connected to an aluminum pointer that moved as an answer to the movement of the eye. 

Moreover, Guy Thomas Buswell built the first non-intrusive eye-trackers. Then, in the 1950s, Alfred L. Yarbus demonstrated that the task given to a subject has a very large influence on the subject’s eye movement. He also demonstrated that the observer’s attention is often drawn to visual elements that do not offer important information. 

In the 1980s, the birth of using eye tracking to answer questions related to human-computer interaction, took place. Recently, eye tracking has been used to study how users interact with different computer interfaces, as the results can lead to changes in design of the interface. 

In addition, Hoffman stated that visual attention is slightly ahead of the eye. However, as soon as attention moves to a new position, the eyes will follow.

In an eye-tracking system, an infrared camera collects the corneal and pupillary light responses from each eye, while a patient is viewing a visual stimulus on a computer monitor, be it an image, a color, or a person. This data allows the direction of gaze to be estimated at any point in time with great spatial and temporal resolution [**8**]. However, for the eye tracking to be as accurate as possible, a calibration is needed.

Eye tracking can be used in market research for a variety of objectives, including product design tests, web page and email communications tests, and marketing communication tests (commercials, listings, leaflets, sponsorships and product positioning). In-depth interviews, focus groups, and surveys are frequently used in conjunction with it [**3**]. During a focus group or an in-depth interview, some important topics or themes are emphasized, and further investigation is conducted by using eye tracking to identify which stimulus has the greatest impact on the consumers. Moreover, in the case of commercials, studies still need to be undergone, to be able to record the emotional and motivation responses of the target groups and the likes or dislikes regarding a medical product or service.

Eye tracking has been explored to evaluate vision in nonverbal patients, such as children, those with neurological impairments, and people who have developmental abnormalities. Its capacities for assessing the severity of strabismus and for visual rehabilitation have also been evaluated [**8**]. The study undergone by Chang focused on children with cortical visual impairment (CVI), as for example, vision loss due to damage to the post-geniculate visual pathways in the brain, this being the most common cause of pediatric visual impairment in the U.S. and developed countries [**8**]. 

There are three types of eye-tracking systems: fixed (as part of a display), mobile (connected in different displays) or wearable (glasses). The video-based eye tracking has become a widely used technique for measuring visual attention and many studies have been published regarding the cause and effect of visual attention in different contexts. To explore attention to social cues in clinical and healthy populations, several eye-tracking research in the social environment have used static stimuli (for example, facial expressions) or dynamic stimuli (for example, brief video clips of social interaction) [**9**]. 

Eye tracking represents the process of estimating the point of gaze or the movement of an eye relative to the head [**10**], the technology enabling the detection of a person’s preference and what they are looking at in real-time. The technology turns eye movements into a data stream containing information regarding pupil position, gaze vector for each eye, and gaze point. Thus, the technology has the role of decoding the eye movements and of translating them into insights that may be used in many fields, such as neuro-ophthalmology or even neuromarketing [**11**]. 

Usually, an eye-tracking system is made up of one or more cameras, light sources and computing capabilities. Basically, algorithms translate the camera feed into data points by using machine learning and advanced image processing, leading to three categories of eye trackers: the first measure the movement of an object attached to the eye, the second allow optical tracking without direct contact to the eye, and the third, allows the measurement of electric potentials using electrodes placed around the eyes, but the main information is collected from the gaze movements.

## Gaze movements

Basically, people think they can get an idea of the meaning of the text they are reading in a single glance, but, in fact, a glaze is needed at each word, or two words, one at a time. Specialists state that this happens because 1-2 degrees of the visual field of the retina is capable of high resolution, and both eye movements should be used to examine each word in the text [**12**]. When an eye movement is impossible, people move their sight by turning their heads. The brains’ propensity for sparse coding may be the cause of the illusion that people can quickly grasp the “gist” of a scene. Thus, people use the central vision to gather visual data from the scene’s “essential” components, but they are supplemented with guesses. 

The eye movements are of two types: slow and fast. Slow movements are specific to pursuit, and fast movements to saccades. Considering that saccades occur between 2 and 6 times per second, the total number of saccades that occur each day can reach several tens or even hundreds of thousands, possibly revealing a patient’s neurological condition.

Even though people perform a lot of saccades during a day, they hardly ever notice their eye movements. However, the movements of the eyes are visible from the outside, being the most “visible” aspect of brain activity. Thus, one can partially guess what someone is “thinking” or their mental state, by only observing the change of gaze. As such, with the examination of fixation patterns, eye tracking enables the direct, objective, and quantitative monitoring of behavior and can reveal which information from a situation is accessible to the brain. Its application in behavioral and neuroimaging investigations has not yet reached its full potential [**13**]. However, it has been concluded that the human emotions and visual attention are highly correlated with practical marketing applications [**14**], namely, the consumer's gaze points on products of interest, may be determined with a remote eye tracker and the outcomes of this analysis may be heat maps and timestamps, which in their turn, may be used for further investigation, or an application for recording user's visual attention on web pages and advertising slides may be implemented by using a sensor for electrodermal activity in response for the consumer arousal [**14**]. 

Thus, it can be concluded that an eye tracker is an instrument that records and evaluates the eye movements of a person, namely, what a person fixates. In addition, it records the periods of time in which the gaze remains longer on a certain object, image or color or the points in which the eyes move from one side to another, in a slow or fast manner. Some experts in eye tracking have emphasized the significance of different gaze movements, as depicted in **Table 1** [**15**]. 

**Table 1:** Types of information records [**15**]

Type of gaze	Explanation
Fixation	Focus images or objects in the fovea, for about 300 msec, during which, the eye remains relatively immobile. Fixation is the action of remaining with the gaze fixed on the same place for a certain amount of time to take in information (read, understand).
Saccadic movements	These are small, rapid, and precise jumps subject to both voluntary and involuntary or induced control, responsible for the recognition and processing of visual information, and associated with head movements. Saccadic movements or saccades are those simultaneous or rapid movements made by the eyes when they move from one point to another in space.
Follow-up	These are slow conjugated tracking eye movements aimed at maintaining fixation on a moving target. The tracking or also considered a visual path is the sum of fixations and saccades.
Restoration of eye torsion	Restoration of eye twisting deals with the rapid closing and opening of blinks, i.e., it is possible to analyze the greater and lesser demand for attention in terms of the object of study.

## Healthcare marketing strategies in using visual elements

In the field of healthcare marketing, it is acknowledged that the most often used strategies to promote health information rely on science, the aim being to encourage positive health behaviors, by improving patient experience and satisfaction. Moreover, to improve patient experience and to obtain loyalty, the visual marketing materials should embed emotional elements in their messages, to establish a connection with the prospective health care consumers, adopt diversity, equity and inclusion. As most healthcare marketing strategies are also about keeping an eco-friendly orientation, medical organizations have started the online promotion of their services.

Most healthcare organizations in the online setting are interested in learning how to improve their online advertising tactics and how customers interpret marketing communications [**16**]. Throughout the past 20 years, a variety of strategies and techniques have been employed to address these issues. They include eye tracking [**17**], biofeedback, face coding, and cognitive neuroscience and psychophysiology. These methods can be used to evaluate marketing initiatives by analyzing nonverbal bodily reactions. The use of these approaches, which are based on neuromarketing, creates countless opportunities for researching how consumers respond to marketing in general and online advertising in particular.

Eye-tracking technology has been applied in different consumer-related fields, and more specifically, in the online research, in advertising effectiveness. More exactly, in Healthcare Marketing, this technique provides accurate information on consumers’ visual attention as gaze fixations and visualization patterns used in advertising [**18**]. 

According to Hamborg KC et al. [**19**], there has been considerable discussion about the best method to assess the effectiveness of online advertisements, which takes into account: a) user behavior; b) user information processing (for example, attention, recognition, and recall); and c) communication-related characteristics that will produce specific attitudes toward the advertisement or affect purchase intent [**20**]. While some authors support the use of heuristic metrics to assess prospective consumer’s behavior, others choose to use experimental data by concentrating on users’ cognitive processing following exposure to online advertising [**21**], eye-tracking methodology [**22**], or self-assessment methodologies including surveys. However, finding trustworthy data on healthcare communication for the development of new methods is currently one of the biggest challenges in consumer research. This occurs because one person is unable to describe his unconscious behavior or because he lies when questioned via a survey or another technique. With the development of technology, the usage of the eye tracking technique has been adopted, allowing for the real-time acquisition of more precise and comprehensive data regarding what the subject views, the trajectory of the gaze, observation time, and even pupil dilation in response to external stimuli.

Therefore, in light of recent developments, it is possible to define eye tracking as a process that enables monitoring and recording of how a person looks at a particular image, in particular, the areas in which he or she fixes their attention, for how long, and in what order they proceed in their visual exploration [**15**]. 

According to the eye tracking research, the health care advertisements must include relevant information in a message, which captures the attention of prospective health care consumers, in addition to being creative and encouraging interaction with them, as illustrated in **Table 2** [**15**].

**Table 2:** Advertising Elements [**15**]

ELEMENTS	CHARACTERISTICS
Bullet	It is characterized by being a phrase that has attractive elements, and that can also summarize, in a few words, the essence of the message.
Header	The main idea should be expressed here. It is important that its structure is not too elaborate or contains many punctuation marks (such as periods and commas), it is also necessary that it is not too short, since, in general, it provides a complete idea of its purpose.
Logo	The logo is usually located in the upper corners. The location of the logo is of utmost importance in advertising campaigns since it shows which organization is responsible for the advertisement.
Slogan	The catchy phrase must allude to a product, and cannot be forgotten. The slogan revolves around the product that is offered and how it performs with people.
Information	This approach should provide information about everything that is being advertised, it will give the customer a better concept and will not have doubts about the product or service that is being offered.
Illustrations	Images are eye-catching for the public, they have the virtue of transmitting a message without the use of words.

## Conclusion

With a typical infrared remote eye tracker, eye tracking can be effectively used to monitor gaze behavior during face-to-face discussions, as well as determine the advertising impact on consumers in the online context. The eye tracking setup can be successfully used in healthcare marketing, as it may enable the improvement of knowledge about the role of visual attention in prospective health care consumers. At the same time, neuromarketing can prevent the waste of money spent on ineffective marketing campaigns. 

**Conflict of Interest statement**

The authors state no conflict of interest.

**Acknowledgements**

None.

**Sources of Funding**

None.

**Disclosures**

None.
